# Effect of *Metarhizium anisopliae* (MetA1) on growth enhancement and antioxidative defense mechanism against *Rhizoctonia* root rot in okra

**DOI:** 10.1016/j.heliyon.2023.e18978

**Published:** 2023-08-12

**Authors:** Afsana Akter Mimma, Tanjina Akter, Md. Ashraful Haque, Md. Abdullahil Baki Bhuiyan, Md. Zahid Hasan Chowdhury, Sharmin Sultana, Shah Mohammad Naimul Islam

**Affiliations:** a Institute of Biotechnology and Genetic Engineering, Bangabandhu Sheikh Mujibur Rahman Agricultural University, Gazipur 1706, Bangladesh; b Department of Plant Pathology, Bangabandhu Sheikh Mujibur Rahman Agricultural University, Gazipur 1706, Bangladesh

**Keywords:** *Metarhizium anisopliae*, Okra, *Rhizoctonia solani*, Biological control, Antioxidant enzyme activities

## Abstract

*Rhizoctonia solani* is an important necrotrophic pathogenic fungus that causes okra root disease and results in severe yield reduction. Many biocontrol agents are being studied with the intent of improving plant growth and defense systems and reducing crop loss by preventing fungal infections. Recently, a member of the Hypocrealean family, *Metarhizium anisopliae,* has been reported for insect pathogenicity, endophytism, plant growth promotion, and antifungal potentialities. This research investigated the role of *M. anisopliae* (MetA1) in growth promotion and root disease suppression in okra. The antagonism against *R. solani* and the plant growth promotion traits of MetA1 were tested *in vitro*. The effects of endophytic MetA1 on promoting plant growth and disease suppression were assessed *in planta.* Dual culture and cell-free culture filtrate assays showed antagonistic activity against *R. solani* by MetA1*.* Some plant growth promotion traits, such as phosphate solubilization and catalase activity were also exhibited by MetA1. Seed primed with MetA1 increased the shoot, root, leaves, chlorophyll content, and biomass content compared to control okra plants. The plants challenged with *R. solani* showed the highest hydrogen peroxide (H_2_O_2_) and lipid peroxidation (MDA) contents in the leaves of okra. Whereas MetA1 applied plants showed a reduction of H_2_O_2_ and MDA by 5.21 and 14.96%, respectively, under pathogen-inoculated conditions by increasing antioxidant enzyme activities, including catalase (CAT), peroxidase (POD), glutathione S-transferase (GST), and ascorbate peroxidase (APX), by 30.11, 10.19, 5.62, and 5.06%, respectively. Moreover, MetA1 increased soluble sugars, carbohydrates, proline, and secondary metabolites, viz., phenol and flavonoid contents in okra resulting in a better osmotic adjustment of diseases infecting plants. MetA1 reduced disease incidence by 58.33% at 15 DAI compared to the *R. solani* inoculated plant. The results revealed that MetA1 improved plant growth, elevated the plant defense system, and suppressed root diseases caused by *R. solani*. Thus, MetA1 was found to be an effective candidate for the biological control program.

## 1 Introduction

Okra (*Abelmoschus esculentus* L.), belongs to the Malvaceae family and is an important vegetable produced worldwide in tropical and subtropical climes [1]. It has around 90% water, 2% protein, 7% carbohydrate, 1.2% sugar, 3.2% fiber, 0.1% fat, and trace amounts of minerals [2]. Okra production is, however, restricted by many abiotic and biotic stresses in tropical areas. Significant biotic stresses are the main barrier to producing optimum okra yield [3]. Plant pathogens and insect pests are the factors limiting the quality and quantity of okra production, with total losses of roughly 35–40% globally [4]. Root rot, charcoal rot, *Fusarium* wilt, powdery mildew, southern blight, and the yellow vein mosaic virus are the most prevalent ailments that affect okra [5,6]. Among them, root rot diseases cause several economic losses and are a serious problem in many areas, including Bangladesh [7].

*Rhizoctonia solani* is a serious soil-borne pathogen that causes root rot, stem canker, and pre- and post-emergence damping-off in both agricultural and horticultural crops. *R. solani* was responsible for the prevalence of root rots, which caused losses of 10–80% in various vegetables [5]. The fungus *R. solani* exists as active mycelium in soil that is adapted to withstand harsh environmental conditions for prolonged periods. It causes the root rot disease complex, which kills plants, by infecting their roots and restricting plant nutrition intake [8]. In response to *R. solani* infection, plants produce reactive oxygen species (ROS) as a defense mechanism. The highest ROS levels were detected in plants when *R. solani* initiated sclerotia production [9]. ROS may operate as second messengers, activating several defenses and scavengers of active oxygen species such as catalase (CAT), peroxidase (POD), ascorbate peroxidase (APX), and Glutathione-S-transferase (GST), all of which decrease the oxidative burst and prevent tissue necrotization [10].

Numerous techniques, including chemical insecticides, nanomaterials, and biological control agents (BCAs), are used to control pathogen infections and decrease crop loss [11,12]. Among them, the use of beneficial fungi and bacteria as BCA is a potential strategy for plant disease control [13,14]. In comparison to chemical pesticides, they do less damage by lowering the chemical risk in the environment. Moreover, BCAs promote plant growth by supplying nutrients and increasing water absorption through altering root morphology and/or rhizosphere interaction [15]. BCAs may also develop resistance to infections by activating hormone-mediated (salicylic acid, jasmonic acid, and strigolactones) plant-defense mechanisms, and they fulfill the needs of commercial markets for maximum chemical residue limitations on fruits and vegetables [[15], [16], [17]].

*Trichoderma*, arbuscular mycorrhizae, ectomycorrhiza, endophytes, yeasts, and avirulent/hypovirulent strains of certain pathogens are examples of BCA fungi [12,[18], [19], [20]]. The entomopathogenic hypocrealean fungus *Metarhizium anisopliae* (Metschn.) Sorokin is found to be very efficient against a range of insect pests, such as ticks, locusts, thrips, and whiteflies [21]. In recent times, the endophytic functions of *Metarhizium* species have been identified, and their involvement in promoting plant growth and mitigating plant diseases has been documented [21]. Several studies have shown that *M. anisopliae* can colonize the roots of different crops and increase the growth of Arabidopsis, tomato, haricot, bean, cassava, and maize [[22], [23], [24], [25], [26]]. For example, the isolates of *M. brunneum*, *M. anisopliae*, and *M. robertsii* showed rhizosphere competency and the ability to stimulate the growth of wheat, corn, and onion, when applied as seed treatments [27]. In addition, *Metarhizium* spp. produce secondary metabolites that have a range of insecticidal, anticancer, antioxidant, and antibacterial effects. *M. anisopliae* showed antifungal activities against the gray mold fungus *Botrytis cinerea* in tomatoes [21]. Root-colonized wheat plants with *M. anisopliae* exhibited dual functions, which involved promoting the growth of wheat and suppressing the occurrence of *Fusarium* head blight disease [20].

It was evident from a previous study that the expression of the *M. anisopliae* chitinase gene CHIT42 in tobacco confirmed resistance against *R. solani* [28]. However, no effort was made to employ *Metarhizium* spp. against *Rhizoctonia* root rot disease as a BCA. Therefore, the present study aimed to evaluate the role of MetA1 in okra growth promotion and root disease suppression caused by *R. solani* and elucidate its role in enzymatic and nonenzymatic antioxidative defense systems in okra.

## 2 Materials and methods

### 2.1 Experimental materials

Fungus strain *M. anisopliae* isolate MetA1 (NCBI accession OQ581920) from our culture collection, and pathogen *Rhizoctonia solani* were used as experimental fungal isolates. Okra (*Abelmoschus esculentus* L.) variety ‘Okra F1’ (a commercial variety) was used as a host plant throughout the experiment. The experiment was conducted in the net house during March–May 2021 (Okra growing season in Bangladesh).

### 2.2 Antagonistic activity of MetA1 against *R. solani*

#### 2.2.1 Dual culture assay

Mycelial disks (5 mm in diameter) were cut from the developing edge of a 4-day-old colony of MetA1 and *R. solani* and put in opposing directions on the edge of a PDA plate. PDA plates amended with Provax-200 (carboxin 5,6-dihydro-2-methyl-1,4-oxathin-3-carboxamide) at a concentration of 200 ppm were included as a positive control. The PDA plates without fungicidal treatment or MetA1 inoculation were used as negative controls. The infected plates were kept in an incubator at 28 °C. The diameter of the fungal radial development in dual cultures was measured after five days when *R. solani* plates in negative controls had filled the plate. Using the method shown by Rajendiran et al. [29], the percent inhibition (PI) of the mycelial growth of *R. solani* caused by the fungal antagonistic activity over the control was computed from three replications.

$$\%\;Inhibition\;of\;growth,L=\frac{X-Y}{Y}\times 100$$

Where, L = Per cent inhibition of radial growth of pathogen (%)

X = Radial growth of the pathogen (mm) in control.

Y = Radial growth of the pathogen (mm) in treatment.

#### 2.2.2 Culture filtrate assay

The cell-free culture (CFC) filtrates of MetA1 were further evaluated for antifungal activity against *R. solani* isolate. MetA1 was cultured in potato dextrose broth (PDB) for five days on a shaker at 120 rpm at 25 ± 2 °C. The broth was filtered through two layers of Whatman filter No. 1, and the supernatants were collected. The collected supernatants were passed through 0.22 μm membrane filters. A volume of 100 μl CFC filtrates was added to the autoclaved PDA medium at rates of 25, 50, 75, and 100%. PDA plates amended with Provax-200 @200 ppm were included as a positive check. Mycelial plugs of *R. solani* isolate from actively growing margins were transferred onto Petri dishes and incubated at 28 °C for five days in an incubator. The inhibition percentage of the test pathogen was calculated as described previously from three replications.

### 2.3 Characterization of MetA1 for plant-growth-promoting and biocontrol traits

#### 2.3.1 Determination of phosphate solubilization activity

Slightly modified Pikovskaya's agar medium (for 1 L,10g glucose,5g Ca_3_(PO_4_)_2,_0.5g (NH_4_)_2_SO_4_,0.2g NaCl, 0.1g MgSO_4_.7H_2_O,0.2g KCl,0.5g yeast extract, 0.002g MnSO_4_.H_2_O,0.002 g FeSO_4_.7H_2_O, and 15g agar) was used for the detection of phosphate solubilization activity. The medium was aseptically transferred to Petri dishes and inoculated with a 5 mm agar disc cut from a 5-day-old MetA1 culture and incubated at 25 ± 2 °C in darkness for 3–5 days [30]. The formation of a clear halo zone around the colony indicates phosphate solubilization activity.

#### 2.3.2 Catalase test

The catalase test was done following the method of Reiner [31]. The MetA1 was inoculated on a PDA medium and incubated at 25 ± 2 °C for 2 days. A needle was used to transfer the fungus from the culture plate to a clean slide containing one drop of 3% H_2_O_2_. The formation of copious bubbles by breaking down the hydrogen peroxide into water and oxygen indicated the presence of catalase.

### 2.4 Pot experiment

#### 2.4.1 Seed priming

Spores of MetA1 were collected from 5-day-old fungal culture and suspended in a Tween-80 (0.05%) solution. Healthy okra seeds were surface sterilized for 15 min with 1% (v/v) NaOCl, rinsed thoroughly 3 times with sterile distilled water, and dried under laminar airflow on sterile paper. Dry okra seeds were dipped in 50 μl of spore suspension (1 × 10^5^ spores ml^−1^) for 24 h for seed priming. For the untreated control, seeds were dipped in a Tween-80 (0.05%) solution.

#### 2.4.2 Seed sowing

Field soil (sandy loam, P^H^ 6.38, 18% organic carbon, 1.07% organic matter, 0.07% N, 0.01% P, and 0.40% K) was used as the growth medium. The soil was autoclaved twice at 24-h intervals at 121 °C and 15 psi for 20 min. MetA1-treated and non-treated okra seeds were sown in plastic pots, maintaining uniform spacing. The plants were kept in a net house and regularly watered.

#### 2.4.3 Preparation of R. solani inoculum

The *R. solani* inoculum was prepared following the modified methods described in Bhuiyan et al. [32]. The wheat grain was boiled for 20 min, then cooled and poured into a 500 ml Erlenmeyer flask, sealed with a cotton plug, and autoclaved. Ten mycelial discs of three-day-old *R. solani* culture were cut from the edge, transferred into each flask and mixed with the autoclaved wheat grains on a clean bench. Then, flasks were incubated at 25 ± 2 °C for 15 days. At 2–3 days intervals all the grains were shaken and incorporated for uniform distribution of fungal mycelia. The colonized wheat grain was air-dried and stored at 4 °C for future use.

#### 2.4.4 Inoculation of R. solani and plant culture

After 15 days of plant growth, the soil was thoroughly mixed with *R. solani* colonized wheat grain inoculum with an equal amount (100 g) in pathogen treated pots with or without MetA1. Soils of control and only MetA1 colonized pots were mixed with an equal amount of autoclaved wheat grains. Thus, the experiment comprised the following treatments: i) Control: neither MetA1 nor *R. solani*; ii) MetA1: *M. anisopliae* inoculated; iii) R. S: *R. solani* treated; and iv) MetA1 + *R. solani*: plant received both MetA1 and *R. solani.* Water was applied to the plants on alternate days.

### 2.5 Determinations

#### 2.5.1 Plant growth parameters

After 28 days after inoculation (DAI), the plants were carefully uprooted and washed. Then, a range of plant growth parameters, such as shoot length, dry and fresh shoot weight, root length, dry and fresh root weight, leaf diameter, and number of fully grown leaflets, were estimated.

#### 2.5.2 Root colonization confirmation

The plant root colonization by MetA1 was confirmed by the visual observation of characteristic mycelial growth in root sections cultured on SDAY medium with antibiotics as per the method described by Parsa et al. [33].

#### 2.5.3 Photosynthetic pigments

*In situ* leaf chlorophyll content was determined by measuring the SPAD (Soil Plant Analysis Development) value (SPAD-502, Minolta Co. Ltd. Japan) weekly, starting one week after pathogen inoculation and continuing until four weeks. Each SPAD value was the average of 10 readings (5 on each side of the leaf midrib) [34]. Chlorophylls (Chl *a*, Chl *b, and* total chlorophyll) and carotenoids’ contents were estimated spectrophotometrically from the 3rd fully expanded leaf sample during harvest using the method described by Porra et al. [35]. Chlorophylls: Chl *a*, Chl *b,* and total chlorophylls and carotenoids contents were estimated from the 3rd fully expanded leaf sample using the method described by Porra et al. [35].

#### 2.5.4 Proline content, total carbohydrate (CHO), and total soluble sugar (TSS)

Fully expanded 3rd leaf samples were collected, and proline was extracted and determined using the proline standard series using the method described by Bates et al. [36]. Total carbohydrate contents and total soluble sugar (TSS) in okra leaf tissues were estimated according to the methods proposed by Dubois et al. [37].

#### 2.5.5 Total phenolic and flavonoid content

Total phenolic and flavonoid contents in okra leaf tissues were estimated according to the methods of Ainsworth and Gillespie [38] and Zhishen et al. [39]. In summary, 0.1g of okra leaf was homogenized with 1.5 ml of 99.8% methanol before centrifugation at 13,000 rpm for 20 min at 4 °C. After that, the same supernatant was used to determine total phenolic and total flavonoid content using various techniques. To calculate total phenolic, 0.2 ml of 10% (0.2 N) Folin-Ciocalteu's reagent was mixed with 0.4 ml of supernatant and left at room temperature for 15 min. After that, 0.8 mL of 700 Mm Na_2_CO_3_ solution was added to this solution and allowed to stand at room temperature for 2 h. Lastly, the absorbance was measured using a Shimadzu UV 1800 at 765 nm. To quantify flavonoids, 0.4 mL of supernatant was mixed with 0.2 mL of 5% sodium nitrite, 0.3 mL of 10% AlCl_3_.6H_2_O, and 1.5 mL of 1 M NaOH. A vortex machine was used to thoroughly mix the mixture. Finally, absorbance at 510 nm was measured.

### 2.6 H_2_O_2_ and membrane lipid peroxidation (MDA)

H_2_O_2_ was estimated according to the method described by Yu et al. [40]. Briefly, 0.5 g of leaf samples were homogenized in 3 mL of 50 mM K–P buffer (pH 6.5) at 4 °C and the homogenate was centrifuged at 11,500×*g* for 15 min. Three mL of the supernatants were combined with 1 mL of 0.1% TiCl_4_ in 20% H_2_SO_4_ (v/v) and kept at room temperature for 10 min. After that, the mixture was centrifuged once again at 11,500×*g* for 15 min. The absorbance of the supernatant was read at 410 nm for calculating H_2_O_2_ content using an extinction coefficient of 0.28 μM^−1^cm^−1^. The level of membrane lipid peroxidation was measured by estimating malondialdehyde (MDA), following the method proposed by Heath and Packer [41].

### 2.7 Antioxidants enzymes activities

For enzyme activity analysis, leaf samples (0.5 g/sample) were crushed in 1 mL of 50 mM ice-cold K–P buffer (pH 7.0), which comprised potassium chloride (100 mM), ascorbate (AsA, 1 mM), β-mercaptoethanol (5 mM), and glycerol (10%; v/v). Homogenized plant material was centrifuged at 11,500×*g* for 12 min38, and the supernatant was collected for enzyme activity estimation [42].

The catalase activity was measured following the method of Hasanuzzaman et al. [43] by monitoring the reduction in absorbance at 240 nm after 1 min due to H_2_O_2_ breakdown. The peroxidase activity was assessed according to the method described by Hemed and Klein [44]. The ascorbate peroxidase and glutathione S-transferase activities were determined using the spectrophotometric method proposed by Hasanuzzaman et al. [43].

### 2.8 Disease incidence

To estimate disease incidence, the okra seeds were grown in seeding trays under similar experimental conditions where every treatment was replicated four times and each replication contained ten plants. After 20 days of plant growth, the soil was inoculated with *R. solani* inoculum following the method described above. The disease incidence was recorded by counting the number of plants that showed typical symptoms, i.e., stem or root lesions [45], after 15 days after inoculation (DAI). The following formula was used to calculate the percent disease incidence:

$$Disease\;incidence\;\left(\%\right)=\frac{Xi}{X}\times 100$$

Where, Xi = Number of infected plants, and X = Total Number of plants.

### 2.9 Statistical analysis

The statistical analysis of the data was conducted through a factorial design using the Statistics 10 software. The means of different treatments were compared using the Least Significant Difference (LSD) test at a significance level of 5%. Additionally, simple data analysis was performed using Microsoft Excel 2010.

## 3 Results

### 3.1 MetA1 showed antagonism against *R. solani* in dual culture assay

**Fig. 1:**
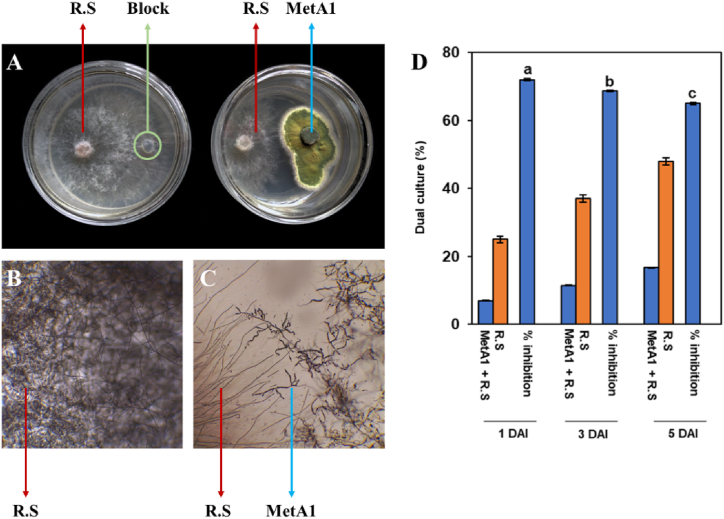
Antagonistic activities of MetA1 against *R. solani* in dual culture assays. (A) Inhibition of mycelial growth of *R. solani* by *M. anisopliae* in dual culture method at 5 DAI. (B) Growth of *R. solani*. (C) Antagonistic activity of *M. anisopliae* against *R. solani* observed under microscope. (D) *In vitro*, antagonistic activities of *M. anisopliae* against *R. solani* in dual culture assays. Values (means ± SEs) with different alphabetical letter above the bars show statistically significant differences (lsd, P < 0.05) among the treatments. MetA1, *M. anisopliae*; R. S, *R. solani*.

In dual culture assays, the endophytic fungus MetA1 inhibited the growth of the virulent pathogenic fungus *R. solani* (Fig. 1A–C). The fungus MetA1 isolate showed antagonistic activity at 1, 3, and 5 DAI by inhibiting radial growth and reducing the mycelial growth of *R. solani* (Fig. 1D). The radial growth of *R. solani* against MetA1 (7.01 ± 0.10 mm) was significantly lower than the control (25.03 ± 0.02 mm) at 1 day after inoculation (DAI), and the percent of growth reduction was observed at 72.04%. Likewise, the radial growth of *R. solani* against MetA1 (11.46 ± 0.07 mm) was significantly lower than the control (37.11 ± 0.11 mm) at 3 DAI, and the percent of growth reduction was 68.7%. Similarly, the radial growth of *R. solani* against MetA1 (16.68 ± 0.12 mm) was significantly lower than the control (48 ± 0.03 mm) at 5 DAI, and the percent inhibition was 65% (Fig. 1D).

### 3.2 Culture filtrates of MetA1 inhibited *R. solani* growth *in vitro*

**Fig. 2:**
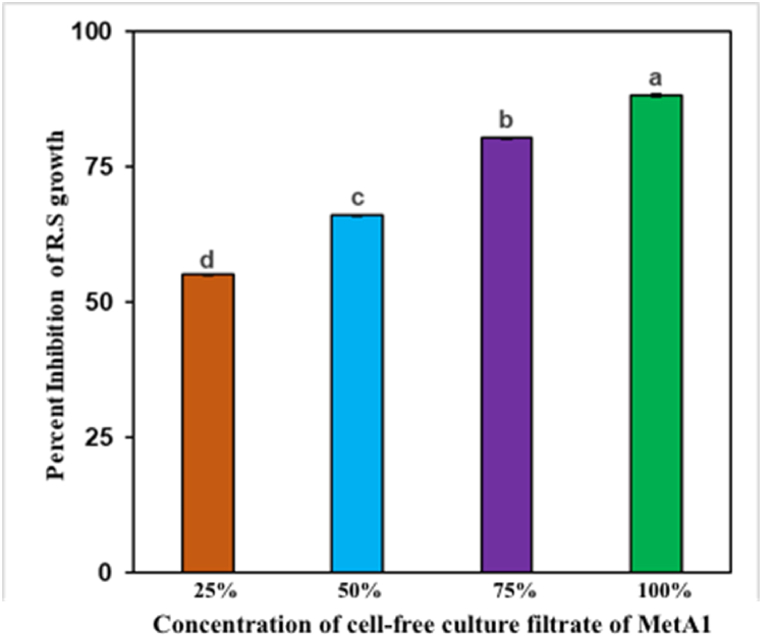
Inhibition of mycelial growth of *R. solani* at different concentrations of cell-free culture filtrate of MetA1. Values (means ± SEs) with different alphabetical letter above the bars show statistically significant differences (lsd, P < 0.05) among the treatments. MetA1, *M. anisopliae*; R. S, *R. solani.*

The cell-free filtrates of MetA1 showed effective antagonism activities against the test pathogen and significantly inhibited the mycelial growth of *R. solani* at all four concentrations compared to the treated control (Fig. 2). The growth inhibition of the test pathogen at 25, 50, 75, and 100% concentrations was 55.18, 66.09, 80.27, and 88.20%, respectively, compared to the negative control on the seventh day after inoculation.

### 3.3 MetA1 showed plant-growth-promoting and biocontrol traits

**Fig. 3:**
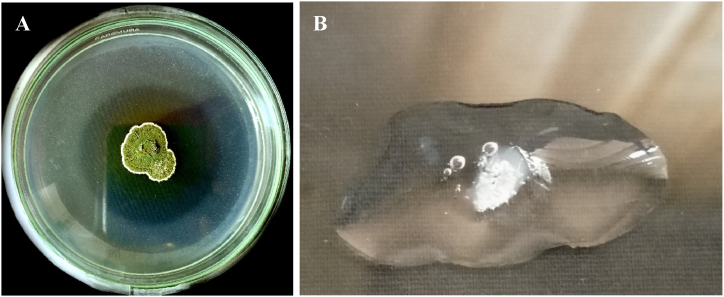
(A) Phosphate solubilization assay of MetA1 in pikovskaya's agar media exhibited strong phosphate solubilize activity and (B) MetA1 exhibited catalase reaction by MetA1 *(M. anisopliae).*

The fungus MetA1 showed strong phosphate solubilization activity in Pikovskaya's (PVSK) agar medium (Fig. 3A**).** In the qualitative estimation of phosphate solubilization, MetA1 showed a clear zone on modified agar after incubation at room temperature for 0–7 days**.** The enzyme assay also showed strong catalase activity by MetA1 by reacting with hydrogen peroxide and forming copious bubbles (Fig. 3B).

### 3.4 MetA1 improved shoot, root, and leaf growth in okra

**Fig. 4:**
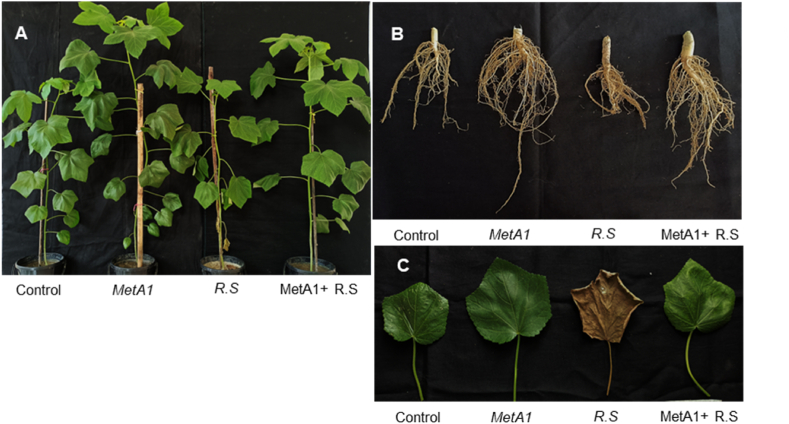
Effects of MetA1 on morphological appearance of the okra plants in a pot experiment. (A) Growth responses of okra plants in different treatments, (B) root morphology, and (C) leaves appearance of okra plants. Where, Control- (without any treatment), Met A1- (*M. anisopliae* inoculation), R.S- *R. solani* inoculation (treated control), and Met A1+ R.S- (*M. anisopliae* + *R. solani* inoculation).

Application of MetA1 in okra showed improved phenotypic appearance by increasing shoot, root, and leaf growth both with and without pathogen conditions (Fig. 4A–C). MetA1-inoculated plants exhibited significantly higher shoot length (5.30%), root length (18.96%), shoot fresh weight (7.48%), root fresh weight (7.56%), shoot dry weight (16.77%), and leaf area (30.45%) as compared to respective control plants (Table 1).

**Table 1:** Effects of MetA1 treatment on growth-promoting traits in okra plants. Values (means ± SEs) of each treatment were attained from four biological replications (n = 4). Mean values with different alphabetical letter(s) denote the statistically significant differences among the treatments, lsd, P < 0.05. MetA1, *M. anisopliae*; R. S, *R. solani*.

Treatment	Shoot length (cm)	Root length (cm)	Shoot fresh weight (g)	Root fresh weight (g)	Shoot dry weight (g)	Root dry weight (g)	Leaf area (cm^2^)	Leaf fresh weight (g)	Leaf dry weight (g)	Number of leaves	Number of full-grown leaflets	Number of flowers	Stem diameter (mm)	Plant height (cm)
**Control**	90.9± 0.75^bc^	16.65± 0.76^bc^	54.292± 0.82^ab^	3.126± 0.06a	9.55± 0.25^b^	0.48 ± 0.02^ab^	97.74± 0.95^c^	39.36 ± 0.98^bc^	5.01 ± 0.06^b^	16.80 ± 0.58^b^	12.20 ± 0.58^ab^	13.00 ± 0.70^a^	6.62 ± 0.07^ab^	80.6± 0.16^ab^
**MetA1**	95.72± 1.41^a^	19.81± 0.43^a^	58.352± 0.92^a^	3.362± 0.13a	11.15± 0.46^a^	0.53 ± 0.03^a^	127.50± 0.69^a^	47.39 ± 1.67^a^	5.64 ± 0.22^a^	19.00 ± 0.44^a^	13.20 ± 0.73^a^	14.60 ± 0.92^a^	6.73 ± 0.09^a^	87.98± 0.21^a^
**R.S**	89.2± 0.84^c^	15.46± 0.23^c^	45.126± 2.66^c^	2.46± 0.07b	6.93± 0.06^c^	0.41 ± 0.01^b^	86.53± 0.27^d^	35.48 ± 1.66^c^	3.79 ± 0.21^c^	15.40 ± 0.67^b^	9.80 ± 0.58^c^	10.40 ± 0.50^b^	5.51 ± 0.22^c^	75.47± 0.67^c^
**MetA1 + R.S**	93.3± 0.75^ab^	17.47± 0.30^ab^	51.106± 0.96^b^	2.726± 0.09b	9.15± 0.02^b^	0.44 ± 0.03^b^	105.77± 1.56^b^	40.85 ± 0.46^b^	4.19 ± 0.23^c^	16.40 ± 0.50^b^	10.40 ± 0.50^b^	12.40 ± 0.50^ab^	6.21 ± 0.09^b^	84± 0.52^b^

Whereas pathogen (*R. solani*) inoculation in plants reduced shoot length (1.87%), root length (7.16%), shoot fresh weight (16.88%), root fresh weight (21.30%), shoot dry weight (27.37%), and leaf area (11.47%) as compared to respective untreated control plants (Table 1). Under pathogen conditions, MetA1-inoculated plants (MetA1+*R. solani*) exhibited significantly higher shoot length (4.60%), root length (13%), shoot fresh weight (13.25%), root fresh weight (10.8%), shoot dry weight (31.94%), and leaf area (22.24%) as compared to only *R. solani* inoculated plants, respectively (Table 1).

In addition, MetA1-inoculated plants exhibited significantly higher leaf fresh weight (20.41%), leaf dry weight (12.61%), leaf number (13.09%), plant height (8.38%), stem diameter (1.69%), full-grown leaf (8.10%), and root dry weight (10.50%) as compared to respective control plants shown in Table 1. In *R. solani* inoculation, plants had reduced leaf fresh weight (9.84%), leaf dry weight (24.31%), leaf number (8.33%), plant height (6.35%), stem diameter (16.73%), full-grown leaf (19.67%), and root dry weight (13.44%) as compared to respective untreated control plants (Table 1). Under pathogen conditions, MetA1+*R. solani*-inoculated plants exhibited significantly higher leaf fresh weight (15.12%), leaf dry weight (10.65%), leaf number (6.49%), plant height (11.20%), stem diameter (12.73%), full-grown leaf (6.12%), and root dry weight (5.82%) as compared to *R. solani* inoculated plants (Table 1).

### 3.5 MetA1 improved SPAD chlorophyll value and photosynthetic pigments

**Fig. 5:**
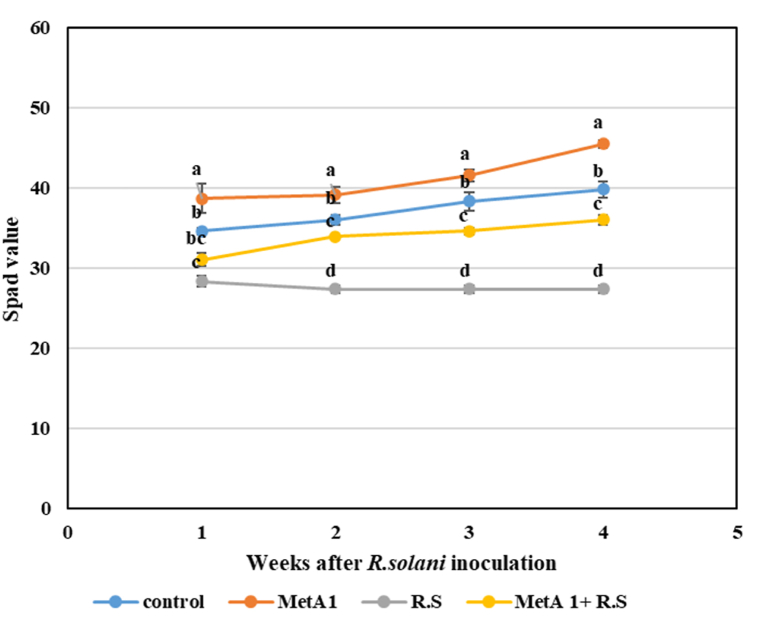
Change of SPAD index after inoculation of MetA1 in okra plant of pot experiment. SPAD reading was recorded in different treatments at 1, 2, 3, and 4 weeks of DAI of *R. solani*. Each point in the line represents the mean value within each treatment category. Values (means ± SEs) with different alphabetical letter(s) denote statistically significant differences (lsd, P < 0.05) among the treatments. Where, Control- (without any treatment), MetA1- (*M. anisopliae* inoculation), R.S- *R. solani* inoculation (treated control), and MetA1+R.S- (*M. anisopliae* + *R. solani* inoculation).

The fungus MetA1 inoculation in okra showed significant improvement in the SPAD chlorophyll value after pathogen infection (Fig. 5). The total SPAD values of the MetA1-inoculated plants in four weeks were significantly higher (11.65, 8.55, 8.0, and 14.22%) as compared to control untreated plants, respectively. Whereas the four weeks’ data showed that, the SPAD values were reduced (18.12, 24.04, 28.64, and 31.23%) in R.S. inoculated plants as compared to untreated controls, respectively (Fig. 5). In biotic stress conditions, MetA1+*R. solani*-inoculated plants exhibited significantly higher values (9.64, 24.04, 26.46, and 31.65%) as compared to the respective *R. solani-*inoculated plants at five time points (Fig. 5).

**Fig. 6:**
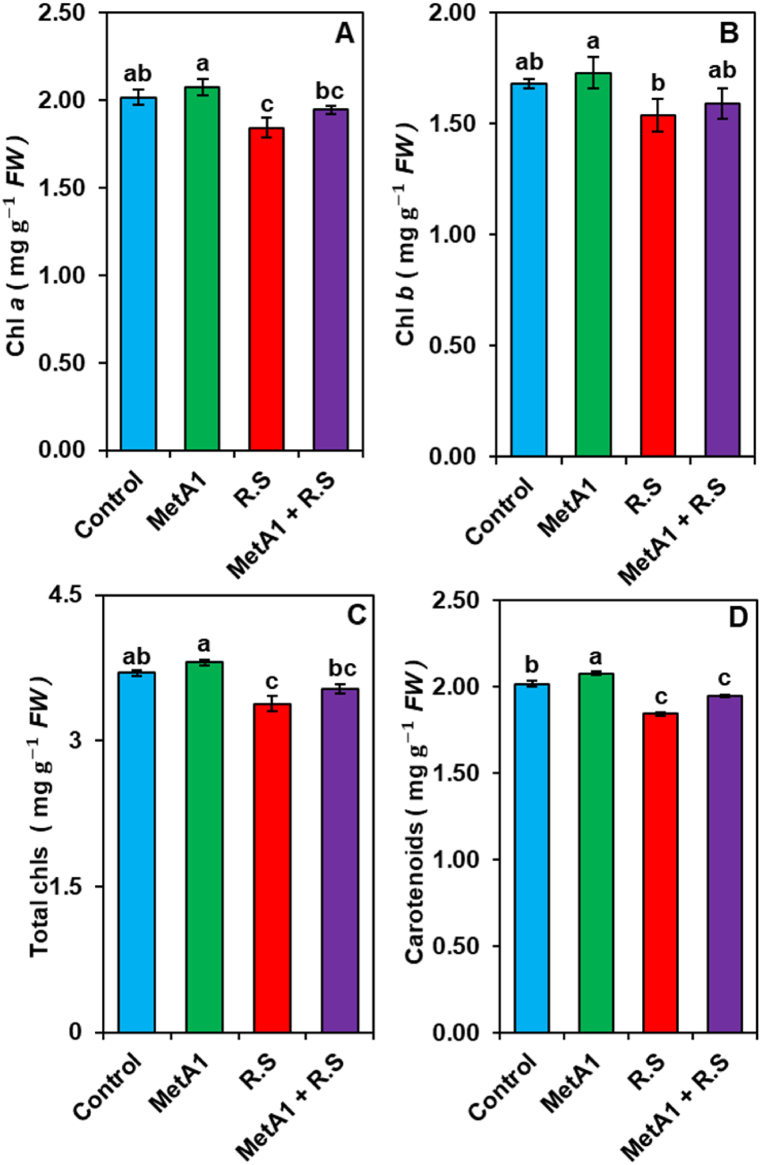
Effects of MetA1 inoculation on the contents of (A) chlorophyll (chl *a*), (B) Chl *b*, (C) total Chls, and (D) carotenoids in leaves of okra plants. Values (means ± SEs) with different alphabetical letter(s) above the bars show statistically significant differences (lsd, P < 0.05) among the treatments. MetA1, *M. anisopliae*; R. S, *R. solani*; FW, fresh weight.

The estimated photosynthetic data showed that MetA1-treated plants increased chlorophyll *a* (chl *a*), chlorophyll *b* (chl *b*), total chlorophylls (chls), and carotenoid contents compared to untreated control plants (Fig. 6A–D). The increased amounts of chl *a*, chl *b*, chls, and carotenoids were observed in plants treated with the endophytic MetA1 strain by 2.92, 2.80, 2.87, and 2.5%, respectively, as compared to their untreated control plants. In stress conditions, *R. solani* inoculated plants decreased chl *a*, chl *b*, total chls, and carotenoids by 8.65, 8.48, 8.57, and 12.43%, respectively, as compared to their untreated control plants (Fig. 6A–D). While MetA1+*R. solani* treatments showed higher chl *a*, chl *b*, total chls, and carotenoids by 5.61, 3.28, 4.55, and 1.84%, respectively, as compared to their pathogen-treated *R. solani* plants (Fig. 6A–D).

### 3.6 MetA1 colonized in okra root

The percent of MetA1 colonization was 44.44 and 38.88% in root samples of MetA1 and MetA1+*R. solani* treatments, respectively. There was no colonization observed in the control and *R. solani-*treated plants.

### 3.7 MetA1 colonized okra plant showed less oxidative damage after *R. solani* inoculation

**Fig. 7:**
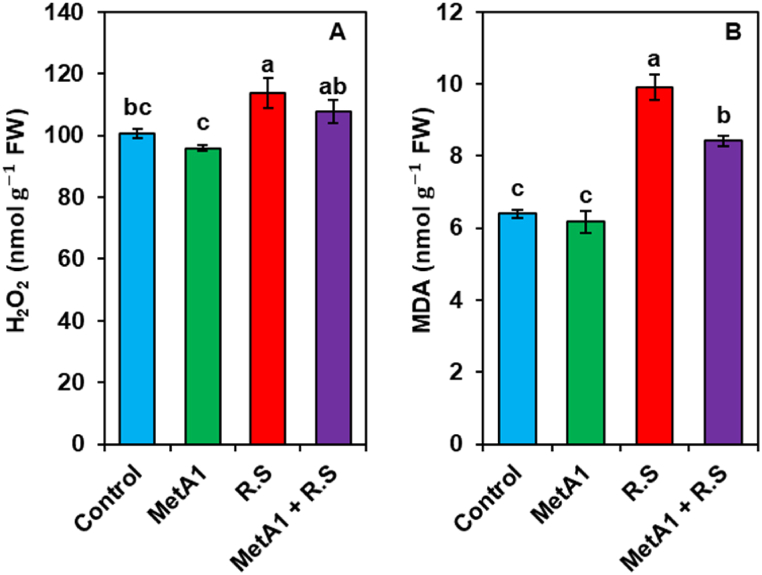
Effects of MetA1 inoculation on the content of (A) H_2_O_2_ content, (B) malondialdehyde (MDA) content in okra plants challenged with *R. solani.* Values (means ± SEs) with different alphabetical letter(s) above the bars show statistically significant differences (lsd, P < 0.05) among the treatments. MetA1, *M. anisopliae*; R. S, *R. solani*; FW, fresh weight.

The *R. solani-*inoculated plants had a profound increase in H_2_O_2_ content (Fig. 7A). Upon exposure to the pathogen, H_2_O_2_ content increased by 12.93% in *R. solani-*inoculated plants compared to untreated control plants. Importantly, a reduction of H_2_O_2_ content was observed in MetA1+*R. solani-*inoculated plants by 5.21% as compared to plants treated with *R. solani* only. The MetA1-inoculated plants also showed a reduced (4.70%) level of H_2_O_2_ as compared to untreated control plants (Fig. 7A).

Similarly, MDA content was increased by 54.73% in *R. solani-*inoculated plants compared to untreated control plants (Fig. 7B). While a significant reduction of MDA content was observed in MetA1+*R. solani* inoculated plants by 14.96% as compared to plants treated with *R. solani* only. The MetA1-inoculated plants showed a 3.51% reduced amount of MDA compared to untreated control plants (Fig. 7B).

### 3.8 MetA1 improved antioxidants levels of okra plants

**Fig. 8:**
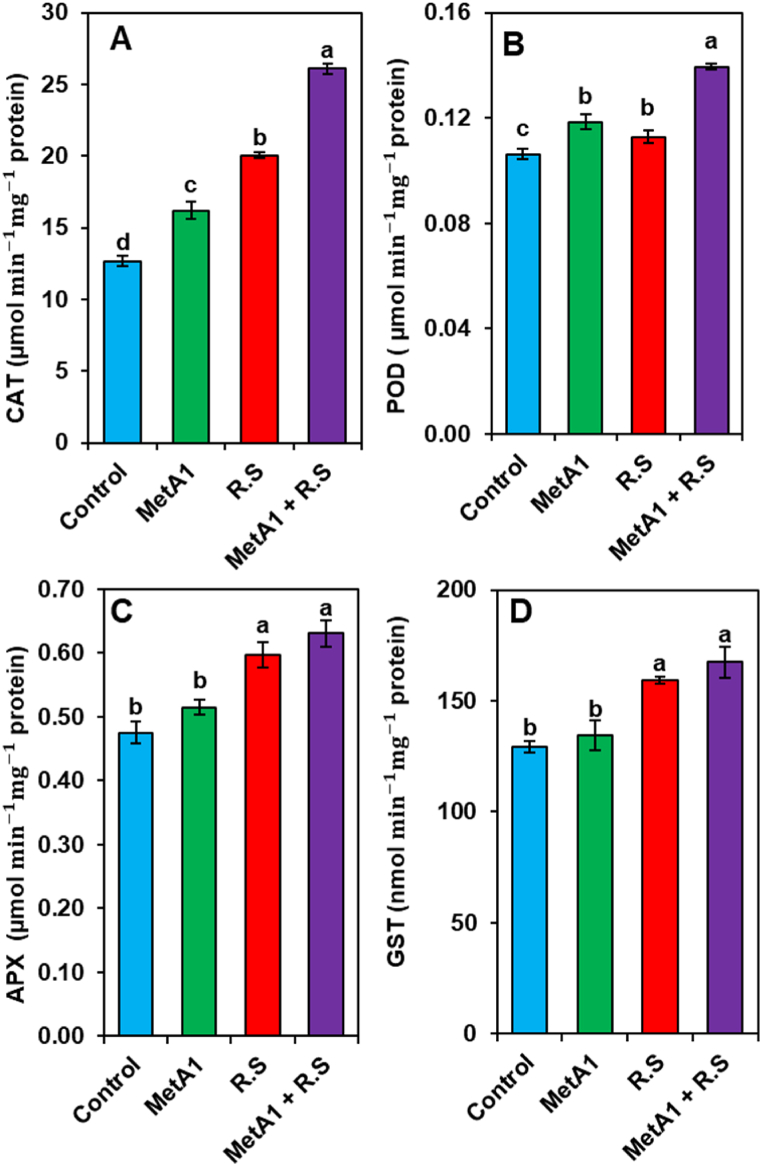
Effects of MetA1 on the activities of (A) catalase (CAT), (B) peroxidase (POD), ascorbate peroxidase (APX), (C) ascorbate peroxidase (APX), and (D) glutathione S-transferase (GST) in leaves tissues of okra plants. Values (means ± SEs) with different alphabetical letter above the bars show statistically significant differences (lsd, P < 0.05) among the treatments. MetA1, *M. anisopliae*; R. S, *R. solani*.

The highest levels of antioxidants (CAT, POD, APX, and GST) were observed in MetA1+*R. solani-*inoculated plants, followed by *R. solani-*inoculated plants (only) and MetA1-inoculated plants (only), and the lowest was in control plants (Fig. 8A–D). In CAT activity, MetA1+*R. solani* treatment exhibited a 30.11% higher amount of CAT as compared to *R. solani-*inoculated plants (Fig. 8A). Both the *R. solani-*inoculated and MetA1-inoculated plants showed enhancements in the content of CAT by 59.21 and 27.96%, respectively, as compared to the untreated control plants (Fig. 8A).

The highest POD activity was observed in MetA1+*R. solani-*inoculated plants, which were 10.19% higher compared to only *R. solani-*inoculated plants (Fig. 8B). Both the *R. solani-*inoculated and MetA1-inoculated plants showed enhancements in the content of POD by 19 and 6.30%, respectively, as compared to the untreated control plants (Fig. 8B).

In the case of APX, MetA1+*R. solani-*inoculated plants exhibited a 5.62% higher amount as compared to *R. solani-*inoculated plants (Fig. 8C). Both the *R. solani-*inoculated plants and the MetA1-inoculated plants showed enhancements in the content of APX by 25.76 and 8.44%, respectively, as compared to the untreated control plants (Fig. 8C).

The GST activity revealed that MetA1+*R. solani-*inoculated plants exhibited 5.06% higher, respectively, as compared to *R. solani-*inoculated plants (Fig. 8D). Both the *R. solani-*inoculated and MetA1-inoculated plants showed enhancements in the content of GST by 23.11 and 3.82%, respectively, as compared to the untreated control plants (Fig. 8D).

### 3.9 MetA1 increased osmoprotectants in okra plants under biotic stress

The highest levels of osmoprotectants (proline, total soluble sugars, and total carbohydrates) were observed in MetA1+*R. solani* plants, followed by *R. solani-*inoculated plants (only) and MetA1-inoculated plants (only), and the lowest was in control plants (Table 2).

**Table 2:** Effects of MetA1 on the level of proline, total carbohydrates, and total soluble sugars in the leave tissues of okra plants. Mean values with different alphabetical letter(s) denote the statistically significant differences among the treatments, lsd, P < 0.05. MetA1, *M. anisopliae*; R. S, *R. solani*; FW, Fresh weight.

Treatments	Proline (μmol g^−1^ FW)	Total carbohydrates (mg g^−1^ FW)	Total soluble sugars (mg g^−1^ FW)
**Control**	0.82 ± 0.08^c^	15.29 ± 0.60^c^	5.13 ± 0.40^c^
**MetA1**	1.02 ± 0.08^bc^	17.18 ± 0.38^bc^	7.17 ± 0.59^b^
**R. S**	1.31 ± 0.03^b^	19.08 ± 0.50^b^	8.72 ± 0.35^ab^
**MetA1+R.S**	1.89 ± 0.16^a^	21.48 ± 0.89^a^	9.78 ± 0.53^a^

The MetA1+*R. solani* treatment significantly exhibited a 44.77% higher proline content as compared to *R. solani-*inoculated plants. Both the *R. solani* and MetA1-inoculated plants had enhanced proline by 60.39 and 25.01%, respectively, as compared to the untreated control plants. In total soluble sugar content, the MetA1+*R. solani*-inoculated plants exhibited significantly higher soluble sugar content (12.15%) as compared to the R. *solani-*inoculated plants. Both the *R. solani* and MetA1-inoculated plants led to an enhancement of sugar by 70.11 and 39.9%, respectively, as compared to the untreated control plants.

The total carbohydrate level data showed that MetA1+*R. solani*-inoculated plants exhibited a 12.64% higher total carbohydrate level as compared to *R. solani-*inoculated plants. Both the *R. solani* and MetA1-inoculated plants led to an enhancement of carbohydrate levels by 24.73 and 12.37%, respectively, as compared to the untreated control plants (Table 2).

### 3.10 MetA1 increased secondary metabolites in okra plants

**Fig. 9:**
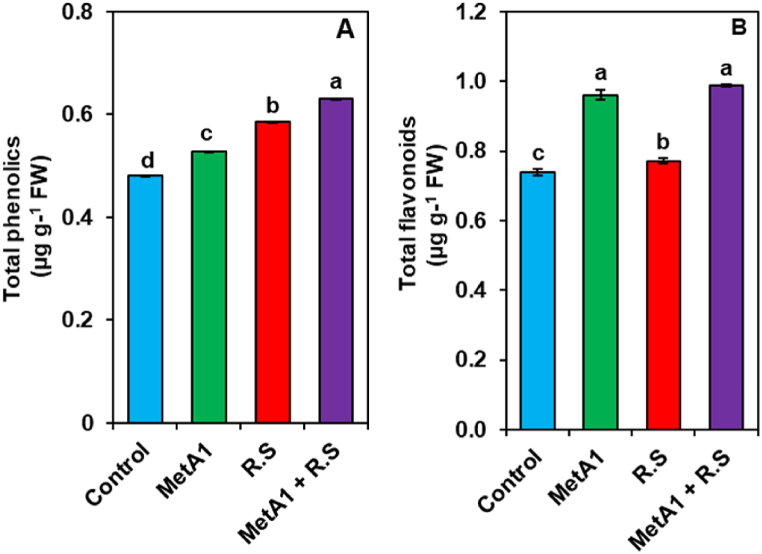
Effect of MetA1 on secondary metabolites of okra plants. The levels of (A) Total phenolics content (B) Total flavonoids contents of leaves tissues of okra plant. Values (means ± SEs) with different alphabetical letter above the bars show statistically significant differences (lsd, P < 0.05) among the treatments. MetA1, *M. anisopliae*; R. S, *R. solani*; FW, fresh weight.

MetA1-inoculated plants showed augmentations in the levels of total phenolics by 1.5% when compared with the untreated control plants (Fig. 9A). *R. solani-*inoculated plants reduced the phenolics level by 2.60% as compared to untreated control plants. In biotic stress conditions, MetA1+*R. solani* inoculation plants exhibited 0.62% higher total phenolic content as compared to *R. solani-*inoculated plants (Fig. 9A). Similarly, MetA1-inoculated plants showed augmentations in the levels of total flavonoids by 10.42% when compared with the untreated control plants (Fig. 9B). In pathogen inoculation, *R. solani-*inoculated plants reduced the total flavonoids level by 22.92% as compared to untreated control plants. In biotic stress conditions, MetA1+*R. solani-*inoculated plants exhibited a significantly higher level of total flavonoids (6.78%) as compared to only *R. solani-*inoculated plants (Fig. 9B).

### 3.11 MetA1 reduced the disease incidence of okra

**Fig. 10:**
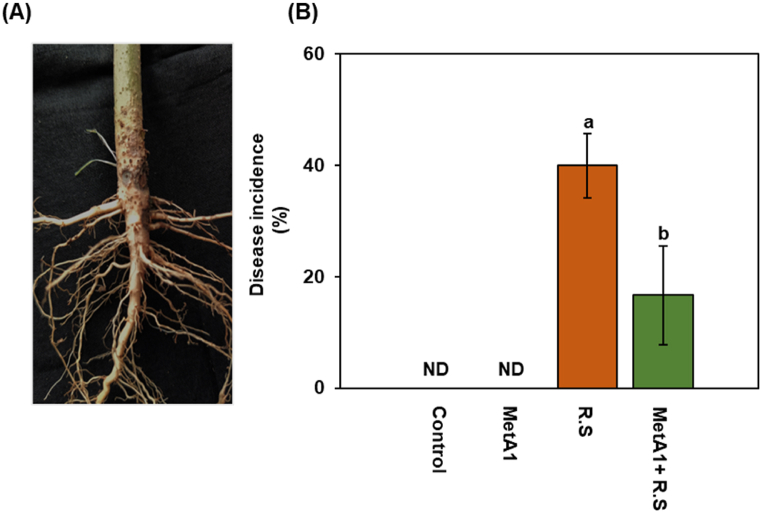
Disease incidence of okra plant root in a pot experiment. (A) Stunted root growth and a darkish spot in the root. (B) MetA1 reduced disease incidence at 15 days after inoculation of *R. solani.* The highest disease incidence was observed in *R. solani* inoculated plants. No disease was observed in untreated control (T1) or MetA1 inoculated plants (T2). Values (means ± SEs) with different alphabetical letter above the bars show statistically significant differences (lsd, P < 0.05) among the treatments. MetA1, *M. anisopliae*; R. S, *R. solani*; ND, no disease detected.

Disease incidence characterized by stunted root growth and darkish spot (Fig. 10A) in only pathogen-challenged plants was 40% at 15 days after inoculation (DAI), which was statistically different from MetA1 inoculated with *R. solani* plants (16.67%) (Fig. 10B). No disease was observed in both untreated control and MetA1-inoculated plants.

Visualization of data with clustered heatmap and unveiling the treatment-variable relationship by PCA.

**Fig. 11:**
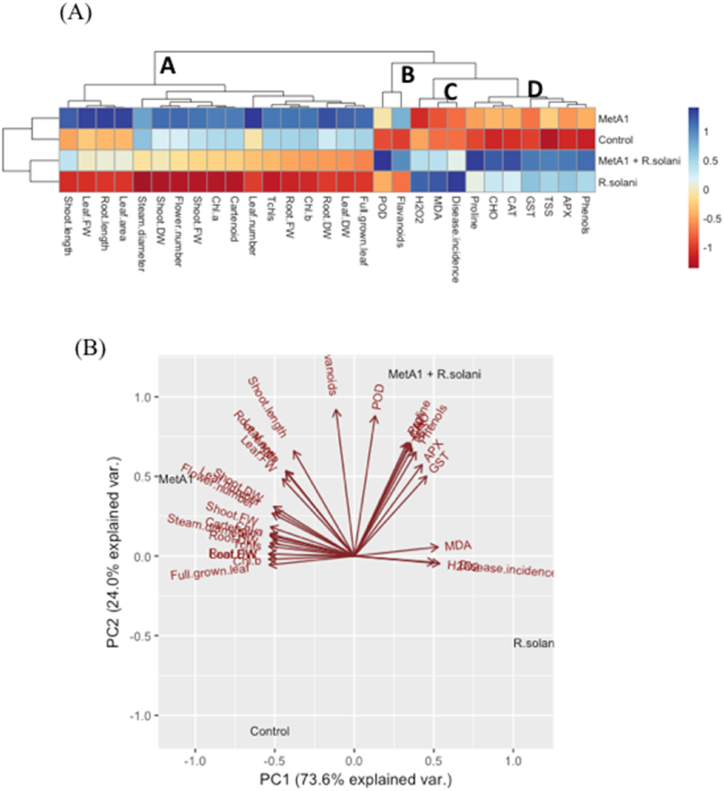
(A) Clustering heatmap visualizing different parameters under different treatments at a glance. Normalized mean values of different parameters were used to prepare the heatmap. The parameters were grouped into four (A–D) distinct clusters. Color scale indicates the changing trend of the normalized mean values of different parameters under different treatments. (B) Principal component analysis (PCA) represents the relationship among the different treatments and parameter. MetA1, *M. anisopliae*; R. S, *R. solani*.

The heatmap utilized color intensity to represent the data, and the parameters were grouped into four distinct clusters using hierarchical clustering (Fig. 11A). This allowed for a better understanding of the relationship between treatments and variables. Cluster A described the growth parameters (shoot length, leaf fresh weight, root length, leaf area, stem diameter, shoot dry weight, shoot fresh weight, chl *a*, carotenoids, leaf number, root dry weight, CAT, leaf dry weight, full-grown leaf, total chls, root fresh weight, chl *b*). Cluster B indicated the antioxidant and secondary metabolites (POD and flavonoid), Cluster C described the stress indicator (H_2_O_2_, MDA, and disease incidence), and Cluster D indicated oxidative stress mediator enzymes and secondary metabolites (APX, GST, CAT, TSS, Total CHO, proline, and phenols). The “control” and “MetA1”-treated plants displayed a positive association with plant growth parameters in cluster A (Fig. 11A). When compared with the “control” plants, the parameters of cluster A exhibited a declining trend in *R. solani-*treated plants (Fig. 11A). The oxidative stress parameters and disease incidence in cluster C exhibited a strong association with *R. solani-*treated stress conditions; however, all the features of this cluster declined upon MetA1 treatment (Fig. 11A). The antioxidants and secondary metabolites in both clusters D and B showed the strongest association with “MetA1+*R. solani,”* followed by *R. solani* treatments (Fig. 11A). In comparison with the “control” plants, the parameters of cluster D displayed an increasing tendency under *R. solani* and “MetA1+*R. solani”* treatment conditions (Fig. 11A).

A principal component analysis (PCA) was performed to determine the relationship between different treatments and variables, as shown in Fig. 11B. The results indicated that PC1 (73.6%) and PC2 (24.0%) accounted for most of the variability, with a total explained variability of 97.6%. Interestingly, cluster A and cluster B parameters were closely associated with the “MetA1” and “MetA1+*R. solani”* treatments, while cluster B and cluster C variables were found to be closely related to the “MetA1+*R. solani”* and “*R. solani”* treatments. Moreover, the “MetA1” plants were found to have a closer relationship with cluster A and cluster B variables than cluster C and cluster D variables (Fig. 11B).

## 4 Discussion

The soil-borne phytopathogenic fungus *R. solani* is widespread across the globe and is commonly managed through the application of chemical fungicides [46]. As a component of Integrated Pest Management (IPM), biological control provides an environmentally beneficial strategy for managing plant diseases. This study evaluated the efficacy of endophyte *M. anisopliae* against plant pathogen *R. solani* through *in vitro* and dual culture antagonistic bioassays; the growth improvement and inhibition of *R. solani* by *Metarhizium* colonized okra plants in a pot experiment; and the role of *Metarhizium* induced antioxidative defense responses against *R. solani* infection in okra plants.

The results of the dual culture assay indicated that MetA1 inhibited the growth of *R. solani* (Fig. 1A). The microscopic examination of the inhibition zone revealed that the growth of *R. solani* had ceased (Fig. 1C). Previous research suggested that the metabolites produced by entomopathogenic fungi inhibit plant pathogen growth [23,47,48]. Using a CFC filtrate bioassay (Fig. 2), this study also confirmed that MetA1 produces antifungal metabolites. This was in line with previous reports, which found that CFCs produced by hypocrealean fungi were effective at inhibiting the growth of fungal pathogens [21,23,49,50]. A recent study found that CFCs of *M. anisopliae* containing active toxic compounds and secondary metabolites damaged the mycelium and conidial plasma membrane integrity of the mold-causing fungus *Botrytis cinerea,* respectively [21]. Besides the production of insect cuticle-degrading secondary metabolites like destruxins, swainsonine, and polyketides [51], *Metarhizium* also produces the secondary metabolites aurovertins, fungerin, N-(methyl-3-oxodec-6-enoyl)-2-pyrroline, and N-(methyl-3-oxodecanoyl)-2-pyrroline, which showed antagonistic effects against *Phytophthora sojae* and *Aphanomyces cochlioides* [52]. A wide range of antibiotics produced by the species *Beauveria, Metarhizium, and Trichoderma* are being approved by the European Union as fungicides and insecticides [53]. However, fungal metabolites produced by MetA1 with fungal inhibition properties require further investigation and the development of biocontrol agents for the prevention of plant diseases.

The plant growth potentiality of microorganisms is associated with different physiological activities like mineral nutrient solubilization, synthesis of phytohormones and enzymes, plant protection against biotic stress, etc. [54]. The isolate MetA1 showed plant growth ability by solubilizing phosphate and producing extracellular catalase enzymes *in vitro* test (Fig. 3A and B). The strains of *M. marquandii* showed the ability to solubilize phosphorus and produce indoleacetic acid (IAA), and their soil inoculation increased plant height, dry weight, and P and N contents in maize, bean, and soybean plants [55]. It has been reported that plant growth-promoting rhizobacteria from the rhizosphere showed similar catalase activity [[56], [57], [58]].

In pot conditions, the fungus MetA1 promoted the growth of okra plants by enhancing photosynthesis, which resulted in increased shoot length, root length, shoot fresh weight, root fresh weight, shoot dry weight, leaf area, leaf fresh weight, leaf dry weight, leaf number, stem diameter, mature leaf, and root dry weight (Fig. 4 and Table 1). The PCA results supported our results by demonstrating that, compared to only pathogen-inoculated plants, MetA1-treated plants under *R. solani*-challenged conditions displayed a stronger positive relationship with plant growth characteristics (Fig. 11B). Endophytic colonization of fungi increased SPAD chlorophyll fluorescence in wheat, lettuce, maize, tomato, and zucchini after the inoculation of *Trichoderma atroviride* [59,60]. The growth promotion of cucumber by *Metarhizium* spp. by increasing the synthesis of chlorophyll and phenolic compounds was also reported [61]. The chlorophyll content was also increased in wheat and cucumber plants treated with *T. longibrachiatum* and *P. indica,* respectively [62,63].

In our study, we observed an increased level of H_2_O_2_ and MDA in pathogen-inoculated plants (Fig. 7A and B). Biotic stress caused a remarkable increase in H_2_O_2_ content, which is a clear indication of oxidative stress [64]. An augmented level of H_2_O_2_ along with enhanced lipid peroxidation is a parameter for the extent of membrane damage that ultimately leads to cell death [[65], [66], [67]]. Plants have developed a strong antioxidant defense mechanism comprising both enzymatic and non-enzymatic antioxidants to counteract oxidative damage caused by ROS under biotic stress. The PCA analysis also demonstrated that MetA1-primed pathogen inoculated okra plants had a negative correlation with ROS products and MDA levels and a strong positive correlation with the activities of enzymatic antioxidants in comparison to *R. solani*-challenged non-inoculated plants (Fig. 11B).

CAT, an enzyme that removes hydrogen peroxide (H_2_O_2_) in peroxisomes, was the first antioxidant enzyme discovered [68]. According to our findings, the CAT activity of okra plants colonized by MetA1 was boosted after being inoculated with the pathogen *R. solani* (Fig. 8A). Similar activity was found when *T. harzianum* T22 colonized plants and modulated the expression of the genes encoding antioxidant enzymes, increasing CAT activity during stress conditions [69]. An increase in the activity of antioxidant enzymes mitigates oxidative damage and rectifies the photosynthetic imbalance resulting from pathogenic lesion production [70].

The stress-related protein POD, which is found in higher amounts when the plant is under stress, is responsible for regulating a variety of processes, including photosynthesis and respiration, as well as the lignification and development of cell walls and the infection of pathogens [71]. In our investigation, MetA1 treatment of pathogen-challenged okra led to a significant increase in POD activity (Fig. 8B). Our findings corroborated previous research indicating that POD is a stress-related protein that increases under stressful conditions [72]. Additionally, inoculation of MetA1 with the pathogen further elevated the activities of APX as compared to pathogen treatment alone (Fig. 8C). This APX employs ascorbic acid as a reducing agent to facilitate the reduction of hydrogen peroxide to water and adds a second layer of protection [68,70]. In similar research, *Trichoderma atroviride* ID20G inoculation ameliorates drought stress-induced damage by improving antioxidant defense in maize seedlings [73].

The enzyme GST protects the cell from oxidative injury by neutralizing reactive molecules with glutathione (GSH) [74]. We observed a higher level of GST in plants that were treated with *R. solani,* and this rose even further in MetA1-colonized okra plants that had been treated with *R. solani* (Fig. 8D). The level of GST increased in okra seedlings damaged by soil-borne *Rhizoctonia*, suggesting activation of the glutathione conjugation system in defense mechanisms counteracting invasion and eliminating the consequences of the infection, which agrees with our results [75].

Soluble sugars and carbohydrates act as important osmolytes, playing multiple functions in plants, including restriction of water loss, stabilization of proteins, and maintenance of osmotic and transcriptional regulation of certain genes. Our study found higher amounts of proline, total soluble sugars, and total carbohydrates in pathogen-challenged MetA1-colonized okra (MetA1+*R. solani* treatment) (Table 2). Proline helps in cell wall lignification via its ability to influence peroxidase activity, which increases the plant's resistance to pathogens [76].

Plants colonized with beneficial fungi and bacteria demonstrate a significant increase in phenolic substances, which induce a plant defense response through changing different metabolic processes [77]. In our study, MetA1-colonized okra plants had increased levels of the secondary metabolites phenolics and flavonoids (Fig. 9A and B). These secondary metabolites, which were shown to be raised to a greater degree in MetA1-colonized plants after *R. solani* infection, provided evidence of a heightened level of plant defense. It was also evident from other studies that the bacterial endophyte *Alcaligenes faecalis*, reprogrammed the host's defense by increasing phenolics and protecting the okra plant from the collar rot fungus *Sclerotium rolfsii* [67]. The combined application of *Trichoderma album* and *Bacillus subtilis* managed *Sclerotinia* root disease by increasing defense metabolites, phenols, and flavonoids in tomatoes [78]. Thus, the current investigation has demonstrated the potential involvement of MetA1 in the regulation of the antioxidant system, leading to the development of an adaptive mechanism that mitigates ROS accumulation and oxidative damage in response to pathogenic stressful conditions.

The disease incidence data revealed that MetA1 reduced disease incidence percent in MetA1+*R. solani* treated plants compared to only *R. solani* treatment (Fig. 10B). Other studies also showed the suppression of plant pathogens by *Metarhizium.* The leaf blight pathogen *Cochliobolus heterostrophus* was suppressed in maize by *M. robertsii* seed treatment [79], and *Fusarium solani* f. sp. *phaseoli* was suppressed in haricot beans by soil application of that species [80]. The reduced occurrence of disease in plants treated with MetA1 and *R. solani* may be attributed to the impact of mycoparasitism or competitive exclusion in the root zone, which helps in the prevention of pathogen invasion [81].

In summary, the application of *M. anisopliae* isolate MetA1 elevated the antioxidative defense system in the okra plant and improved the activities of antioxidants by increasing different enzyme activities, viz., CAT, POD, GST, APX, and nonenzymatic antioxidants, viz., phenol and flavonoid, which were further elevated after *R. solani* infection. The fungus *M. anisopliae* also increased soluble sugars and carbohydrate contents, indicating a better osmotic adjustment in diseases infecting okra plants. Thus, MetA1 was found to be an effective candidate for the biological control program.

## 5 Conclusion

The fungal isolate MetA1 improved okra plant growth parameters and was also found effective to reduce *Rhizoctonia* root disease incidence and pathogenic stress by modulating the activity of antioxidant defense enzymes and elevating the nonenzymatic osmoprotectants levels in okra. Therefore, the application of *M. anisopliae* (MetA1) to okra would be a successful strategy for both growth promotion and disease control programs.

## Declarations

### Author contribution statement

Shah Mohammad Naimul Islam and Afsana Akter Mimma: Conceived and designed the experiments.

Shah Mohammad Naimul Islam, Md. Abdullahil Baki Bhuiyan and Md. Ashraful Haque: Contributed reagents, materials, analysis tools or data.

Afsana Akter Mimma, Md. Zahid Hasan Chowdhury, Tanjina Akter and Sharmin Sultana: Performed the experiments, Analyzed and interpreted the data.

Afsana Akter Mimma, Shah Mohammad Naimul Islam and Md. Abdullahil Baki Bhuiyan: Wrote and revised the paper.

### 5.1 Data availability statement

Data associated with this study has been deposited at NCBI accession OQ581920.

### Additional information

No additional information is available for this paper.

## Declaration of competing interest

The authors declare the following financial interests/personal relationships which may be considered as potential competing interests:

Shah Mohammad Naimul Islam reports administrative support and equipment, drugs, or supplies were provided by Bangabandhu Sheikh Mujibur Rahman Agricultural University. Shah Mohammad Naimul Islam reports a relationship with Bangabandhu Sheikh Mujibur Rahman Agricultural University that includes: employment.
